# Artificial Intelligence Readiness in Emergency Medicine: Expert Consensus Opinion for Preparing the Workforce

**DOI:** 10.1016/j.acepjo.2026.100503

**Published:** 2026-09-11

**Authors:** Debadutta Dash, Joyce Macalalad, Donald L. Lum, Mark W. Baker, William C. Dalsey, Rohit B. Sangal, John R. Dayton, Tayab C. Waseem, Neal K. Sikka, Andrew L. Chu

**Affiliations:** 1 Department of Emergency Medicine, Stanford University School of Medicine, California, USA; 2 Department of Emergency Medicine, Northfield Hospital + Clinics, Northfield, Minnesota, USA; 3 Department of Emergency Medicine, University of Hawaii John A. Burns School of Medicine, Honolulu, Hawaii, USA; 4 Department of Emergency Medicine, Capital Health Regional Medical Center, Trenton, New Jersey, USA; 5 Department of Emergency Medicine, Yale School of Medicine, New Haven, Connecticut, USA; 6 Department of Emergency Medicine, George Washington University School of Medicine and Health Sciences, Washington, DC, USA

**Keywords:** artificial intelligence, emergency medicine, clinical decision support, medical education, AI governance

## Abstract

The rapid integration of artificial intelligence (AI) into emergency department workflows has outpaced clinician training, vendor evaluation infrastructure, and specialty-wide governance. Emergency physicians increasingly encounter AI tools spanning triage, imaging, clinical decision support, documentation, and operations, yet most lack the foundational skills to critically evaluate or safely oversee these products. Without a unified framework, departments struggle to distinguish safe tools from risky ones, and no national standard exists to guide education, vetting, or implementation. This expert consensus opinion, an official work product of the American College of Emergency Physicians (ACEP) AI Task Force formally endorsed by the ACEP Board of Directors in 2025, proposes 3 coordinated priorities to address these gaps. Educate: outlines a standardized AI education framework spanning residency training through continuing medical education, anchored in existing specialty-specific competencies. Evaluate: proposes an expert-derived, structured, clinician-led three-stage framework for vetting AI tools and industry partners prior to deployment. Advise: calls for the establishment of a national Emergency Medicine AI Advisory Council to issue shared terminology, best-practice guidance, and implementation toolkits across academic, community, and critical access settings. Together, these recommendations provide an adaptable, expert-derived foundation for ensuring that AI integration in emergency medicine is safe, equitable, and clinically effective.

## 1 Background

In emergency medicine, leading artificial intelligence (AI) use cases are triage and early risk stratification,^1^, ^2^, ^3^ imaging triage and decision support,^4^, ^5^, ^6^ disposition prediction,^7^, ^8^, ^9^, ^10^, ^11^ operations,^12^^,^^13^ time-sensitive disease detection,^14^^,^^15^ documentation and patient communication,^16^, ^17^, ^18^ and billing optimization.^19^ Most AI tools remain in research, pilot, or validation phases, yet deployments are accelerating^20^, ^21^, ^22^, ^23^, ^24^, ^25^ while frontline training lags. Residents and attending physicians often lack the skills to evaluate or oversee these products,^24^^,^^26^, ^27^, ^28^, ^29^ even as startups and established vendors market unproven solutions to emergency departments. Without a shared framework, departments struggle to distinguish safe tools from risky ones,^30^, ^31^, ^32^ and no unified national voice exists to guide education, standards, or vetting, leaving efforts fragmented.^33^, ^34^, ^35^, ^36^

## 2 Objective

To propose best practices for preparing emergency physicians and their teams for the implementation and delivery of care using AI, including the clinical, technical, and workforce competencies needed to safely and effectively integrate AI into emergency medicine practice.

## 3 Approach

This expert consensus opinion represents an official work product of the American College of Emergency Physicians (ACEP) AI Task Force and was formally endorsed by the ACEP Board of Directors in 2025. Developed through structured deliberation among task force members, it proposes best practices for preparing emergency physicians and their clinical teams for the integration of AI into emergency care. The recommendations reflect the collective judgment of emergency physicians actively engaged in AI governance and implementation.

For the purposes of this document, “AI” encompasses any software system that leverages data to automate or augment clinical or operational decision-making, including imaging analysis, triage algorithms, and large language models applied to documentation and communication. The principles outlined here are intended to apply across this full spectrum of AI applications in emergency medicine.

**Figure 1:**
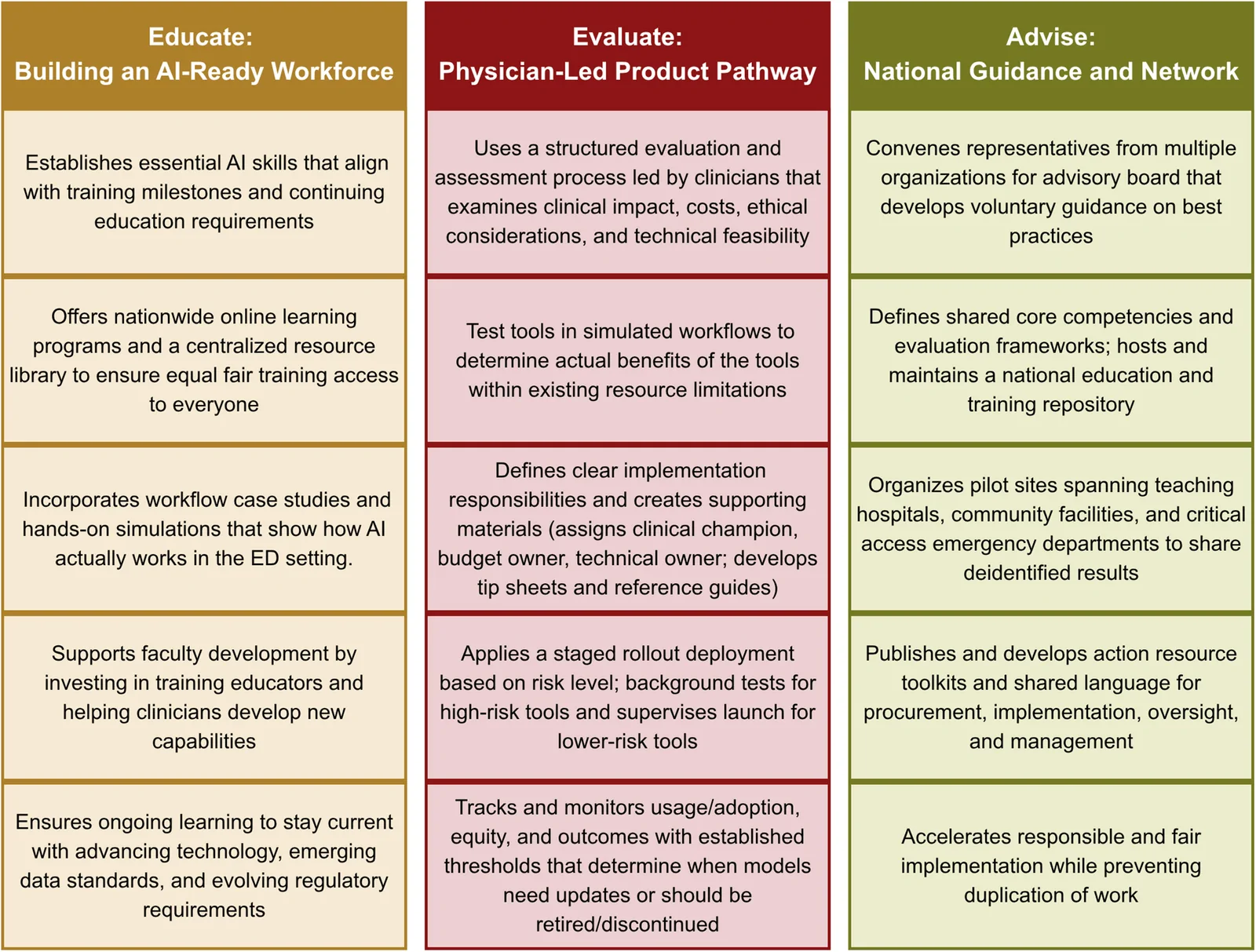
The Educate, Evaluate, and Advise Framework for AI Readiness in Emergency Medicine.

To address these gaps, this expert consensus proposes 3 coordinated priorities for AI readiness in emergency medicine (Fig 1):

• Educate: Establish best practices for clinician education and workforce training.
• Evaluate: Provide an expert-derived framework for assessing and vetting AI tools.
• Advise: Advance a national strategy for the safe, equitable, and effective integration of AI into patient care.

This figure summarizes the 3 pillars of the framework: Educate, which establishes AI literacy and competency standards across the emergency medicine workforce; Evaluate, which provides a structured, clinician-led process for assessing AI tools across clinical, financial, ethical, and technical dimensions; and Advise, which coordinates national guidance, aligns standards, and supports equitable adoption across diverse practice settings.

### 3.1 Educate: Leveraging Existing Resources and Modules

The Educate pillar aims to establish a shared foundation of AI literacy across the emergency medicine workforce. Formal longitudinal AI teaching is uncommon,^26^, ^27^, ^28^^,^^36^, ^37^, ^38^, ^39^ and limited curricular time, scarce faculty expertise, and competing priorities leave a readiness gap.^27^, ^28^, ^29^^,^^36^, ^37^, ^38^, ^39^ Despite these shortcomings, more and more AI-based solutions are being integrated into emergency department (ED) workflows,^22^^,^^23^^,^^38^^,^^40^ and this mismatch in foundational knowledge and practice realities raises concern about patient safety.^20^^,^^22^^,^^23^^,^^27^^,^^38^^,^^40^ The AMA notes physicians may be liable for negligent AI selection, and legal standards are rapidly evolving to include AI use.^34^^,^^41^ This highlights the specialty-wide upskilling needed to keep pace with medico-legal and technical change.^26^^,^^28^ Fortunately, the Association of American Medical Colleges (AAMC) and multiple specialty societies have found consensus on the need for widespread AI literacy across medical training.^29^^,^^34^ The AAMC has defined its own principles for the responsible use of AI in education, and it strongly encourages residency programs to develop comprehensive curricula and core competencies to prepare their workforce to leverage AI effectively.^27^^,^^29^

#### 3.1.1 What exists: a foundation to build upon

Foundational resources for AI education already exist within medicine. Several major medical organizations have developed free, high-quality curricula, including the AAMC’s “AI Skill Building for Medical Educators” webinar series and the AMA’s “Artificial Intelligence in Health Care” micro-learning series.^29^^,^^42^ Together, these programs provide accessible entry points for clinicians to develop AI literacy and suggest that structured AI education in emergency medicine is both feasible and scalable. However, implementation remains fragmented, adoption is inconsistent, and access is often concentrated within well-resourced academic centers.^27^, ^28^, ^29^^,^^39^

#### 3.1.2 What we propose: a Unified National Strategy

We recommend that ACEP, SAEM, CORD, EMRA, AAEM, ACOEP, ABEM, and the ACGME collaborate to build a unified national strategy for AI education and an AI-ready workforce in emergency medicine.^27^^,^^29^^,^^36^, ^37^, ^38^, ^39^^,^^43^^,^^44^ Specifically, this strategy should be grounded in AAMC specialty-specific AI competencies, with 3 core objectives: defining clear ethical and professional guidelines for ED use, establishing a unified evidence-based curriculum for emergency physicians, and embedding foundational skills—algorithm appraisal, bias mitigation, and data governance—into residency milestones and continuous medical education requirements (CME) requirements.^27^^,^^29^ To ensure broad adoption across diverse training environments, the strategy should account for differences in faculty expertise, protected teaching time, informatics support, and institutional infrastructure. Smaller community programs with limited local AI expertise or informatics support could use free, EM-specific virtual modules developed by national organizations and large academic medical centers; programs with moderate capacity could participate in regional workshops; and large academic medical centers and other well-resourced institutions should openly share curricula, implementation guides, and evaluation tools.^27^ ACEP and SAEM can further supplement this foundation with workshops on advanced concepts, hands-on training, and indepth case studies.^27^

### 3.2 Evaluate: A Clinician-Led Framework for Vetting AI Technology

#### 3.2.1 The need for a vetting framework

The Evaluate pillar recommends a structured, clinician-led process for assessing AI tools prior to clinical deployment. The three-stage framework draws on existing evaluation frameworks and the collective clinical and operational experience of the task force, and includes due diligence on AI partners, assessment of clinical value and workflow fit, and workflow design and pre-deployment preparation. Emergency physicians who develop a strong understanding of AI principles and gain hands-on experience through workflow simulation case studies can learn and apply clinician-led frameworks for vetting AI technology in the emergency department.^30^^,^^31^^,^^33^ This will help the arduous process of investing significant time and human capital in interviews and negotiations, grant writing, contracting master service agreements, vetting data security and architecture, and engaging necessary stakeholders only to discover later that the company can’t meet either the technical needs or financial commitment needed to support both the integration process and long-term support.^30^^,^^32^^,^^39^ Without proper assessment of a potential partner’s readiness and capabilities, departmental investments are at risk of becoming wasted efforts.^24^^,^^30^^,^^45^ A structured, clinician-led framework is crucial for guiding prospective internal or external, industry partners through clearly defined developmental stages, utilizing rigorous clinical evaluation methods to ensure that only the solutions that address the most important strategic priorities move forward to an actualized partnership.^29^^,^^31^^,^^33^^,^^46^ Without adequate physician input into the design process, AI tools may not address the most pressing clinical needs.^47^

**Figure 2:**
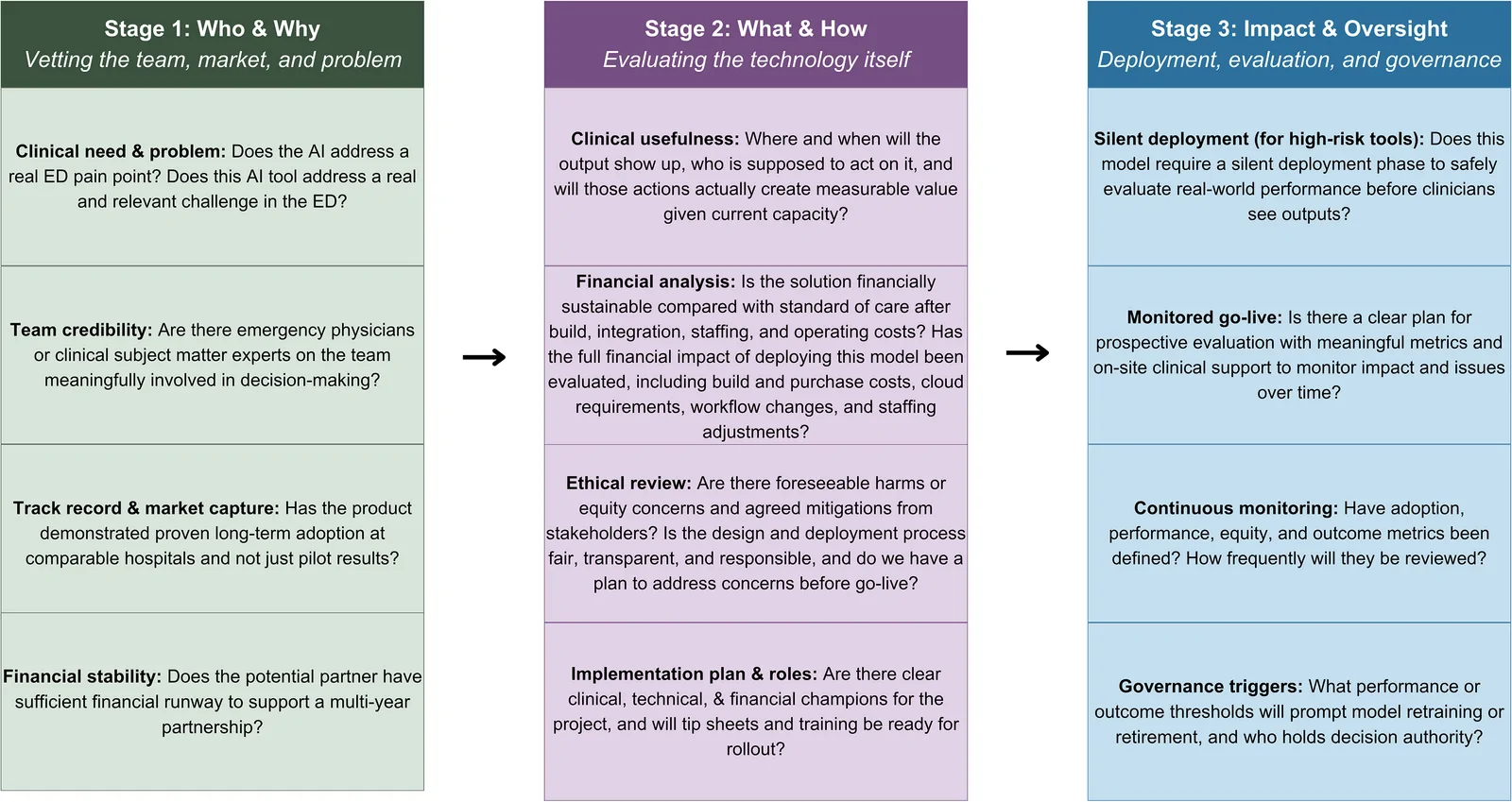
Three-Stage Framework for Vetting and Deploying AI in Emergency Medicine.

This framework guides clinicians through a sequential evaluation spanning 3 stages: Stage 1 (Who & Why) assesses the AI partner, market viability, and clinical problem; Stage 2 (What & How) evaluates the technology across clinical, financial, ethical, and technical dimensions; and Stage 3 (Impact & Oversight) governs deployment, ongoing monitoring, and iteration (Fig 2). Each stage serves as a sequential gate; failure to meet criteria halts advancement until deficiencies are addressed and criteria are met, and culminates in a structured report to support transparent, accountable decision-making regarding AI adoption in emergency care.

### 3.3 Due Diligence on AI Partners

Due diligence starts by asking, “Does this AI tool solve a real, unaddressed ED problem?” Clinicians confirm the solution targets real ED challenges, including faster critical diagnoses, better throughput, or similar priorities.^30^ This early screening helps filter out the technologies that do not align with the department’s top strategic priorities, and helps clinicians to identify which solutions are worth further consideration.^30^, ^31^, ^32^ Academic institutions, such as Stanford, have demonstrated that structured internal pathways for connecting external industry partners with AI and informatics experts can streamline the evaluation of emerging health technologies.^33^

If so, the ED should run a comprehensive due diligence process on both the team and technology. Although the following examples are specific to companies exploring academic or community hospital partnerships, the same principles apply to solutions developed internally within a health system.

#### 3.3.1 Clinical credibility

Ideally, there should be an emergency physician or physician on the founding team who understands the realities and workflows of the ED.^33^^,^^47^ Teams composed entirely of engineers who have never worked in a hospital often miss the stakeholders and nuances required for a successful solution.^24^^,^^33^ Clinical champions are most effective when they bring both frontline clinical expertise and sufficient technical fluency to critically appraise AI tools, evaluate their fit within existing workflows, anticipate integration challenges and errors, and determine whether they address the right use case.^24^^,^^30^ They can provide decision-making authority on matters related to clinical decisions, patient safety, and departmental workflows, as well as early feedback on their experiences with the user interface and outputs of the AI tools.^33^

#### 3.3.2 Track record & experience

Do the founders, product leads, or internal team members have prior experience developing health technology and navigating regulatory requirements, or are they new to the clinical and operational demands of this space? Teams that have previously taken an AI product through the full development and deployment lifecycle in a clinical environment are better positioned to anticipate the operational, technical, and regulatory challenges of a sustained implementation.^30^ Teams should also demonstrate fluency with FDA regulations for AI and ML for past and current products.^32^^,^^48^^,^^49^ For nongenerative AI, single purpose tools such as clinical decision support and predictive algorithms should be classified as Software as a Medical Device (SaMD) when applicable, with clear intended use cases, representative training data, and Predetermined Change Control Plans (PCCP) for safe updates and monitoring across the entire product lifecycle.^31^ For generative AI, which can create new text and images for a wide variety of tasks, the team should have safeguards in place to ensure transparency, bias reduction, and accuracy of all outputs, including mechanisms to monitor and suppress hallucinations or require evidence-backed responses.^24^

#### 3.3.3 Financial stability

Whether a hospital plans to (1) co-develop and validate or (2) pilot and integrate an AI product, this process can take many months to years. It is critically important for partnering hospitals to ensure that any industry partner has the financial runway to support a multi-year partnership.^30^^,^^33^ Startups range in size and funding, from pre-seed (typically <$500K in funding) to Series C and beyond (typically $30M–$100M+), with higher series reflecting longer runways and product maturity. Inadequate assessment of a potential partner’s financial sustainability risks committing substantial clinical and technical resources to a partnership that may ultimately fail.^32^^,^^45^

#### 3.3.4 Market capture

How many peer hospitals of similar size, volume and acuity already pay to use the product? Proven uptake and success at peer institutions can be powerful signals of future performance.^30^^,^^31^ Additionally, it is important for the external partner to meaningfully differentiate itself from competitors attempting to solve the same problems. Preference should be given to vendors that have demonstrated expansion from pilot phase to enterprise deployment with contract renewals and sustained use.^33^^,^^50^

## 4 Proving Clinical Value and Workflow Fit

Although it is important for a company to pass a clinician-led due diligence gateway, it is equally important for the company’s AI product to be evaluated on whether it can actually deliver measurable value at scale.^30^^,^^31^^,^^45^ This deeper product analysis focuses on a structured, clinician-led assessment of the proposed AI workflow itself.^31^^,^^51^ This process is informed by the FURM framework, which describes the relationship between model predictions, the clinical protocol that translates those predictions into clinical decisions, and the capacity of frontline staff to act on those decisions.^52^ Before adopting an AI solution, emergency departments must weigh the financial, ethical, and workflow realities that determine whether a promising concept will translate into real-world clinical benefit.^24^^,^^30^^,^^31^^,^^49^^,^^53^

### 4.1 Clinical Usefulness

Emergency physicians must test vendor claims by mapping the exact workflow step where recommendations surface and judging whether those outputs are actionable amid real-world constraints, including staffing ratios, wait times, CT queues, boarding, and alert fatigue.^12^^,^^15^^,^^30^^,^^31^^,^^53^ Simulation exercises help reveal whether the projected gains are meaningful; if not, ED leaders can move on before investing more time.^30^^,^^31^^,^^54^

### 4.2 Financial Projections

Departmental leaders need to evaluate the financial impact of model deployment.^30^^,^^45^ Financial analysis is performed to ensure the AI tool represents a sustainable investment, factoring in all pertinent expenditures related to building or purchasing the solution.^39^^,^^45^ These expenses include cloud computing requirements, changes to clinical and technical staffing, and costs related to adapting clinical operations and workflows. This information can be used to calculate the differential in anticipated revenue and expense between standard of care and model-guided care.^30^

### 4.3 Ethical Review

Ethical evaluation is equally important at this stage.^24^^,^^31^^,^^49^ Departmental leaders need to take into consideration the perspective of diverse stakeholders, including fellow physicians, nurses, patients, underrepresented communities, and technical staff, with the goal of identifying potential ethical conflicts or oversights in model design and deployment.^20^^,^^24^ Clinicians need to be able to identify potential conflicts of interests and signs of algorithmic bias that could exacerbate health disparities for vulnerable populations.^24^^,^^29^ This ensures the entire process of incorporating AI technology is fair, transparent, and responsible.^24^^,^^49^ If any concerns arise, the affected stakeholders can work together to address these issues prior to deployment.^24^^,^^39^

### 4.4 Workflow Design & Predeployment Preparation

Once a tool clears clinical and financial due diligence, clinicians codesign the deployment plan with IT teams to ensure the AI output appears in the right workflow step and prompts the intended action without disrupting care.^1^^,^^2^^,^^53^ This guidance prevents disruptions to patient care and ensures the tool supports rather than complicates decision-making.^53^

During this phase, emergency physicians become clinical champions, utilizing implementation science best practices, and convening with nurses and ancillary staff to codesign workflows that account for operational hurdles.^15^^,^^24^^,^^39^ These champions provide practical tip sheets and reference guides to standardize use and minimize cognitive burden. Initially, they should also be present on-site for troubleshooting and coaching sessions.^15^ Daily debrief sessions ensure continuous learning and recalibration of the tool and associated workflow, thereby improving real-time performance and iterative feedback.^53^

### 4.5 Continuous Monitoring and Iterative Improvement

After deployment, clinicians measure the tool’s actual impact and continue monitoring.^31^ Physicians help establish clinically meaningful outcomes that go beyond statistical measures, ensuring that with thorough assessment, the evaluation focuses and prioritizes on what truly matters for patient care, ED operations, and clinical outcomes.^30^^,^^47^ At this stage, the physician, as clinical champion, provides ongoing qualitative and quantitative feedback on how the tool influences clinical decisions, team dynamics, and potential problems such as alert fatigue.^15^^,^^24^^,^^53^ They act as the frontline monitor for system performance integrity, understanding how the tool behaves over time.^30^^,^^31^ High-risk tools may undergo silent deployment, running in the live electronic health record with hidden outputs, to observe performance before exposing clinicians, while lower-risk pilots can use shorter silent trials to gather telemetry without disrupting care.^14^^,^^24^^,^^31^^,^^32^^,^^46^

### 4.6 Training Clinicians in AI Evaluation

Successful implementation requires physicians be ready to apply the framework by leveraging a shared online hub with training materials and foundational content on AI in medicine.^26^^,^^29^ Short, online case studies based on real academic-industry partnerships can help physicians develop the skills needed to evaluate potential partners.^24^^,^^31^^,^^32^ Modules may include evaluating vendor simulations, questioning financial projections and sustainability, and recognizing the ethical risks and algorithmic bias affecting the ED population.^24^ Through regular practice, physicians learn to focus on what really matters: evidence, functionality, and clinical applicability of AI tools in the ED environment.^30^^,^^31^ This enables informed decisions about whether a proposed tool has the supporting clinical justification, staffing, and protective measures to turn statistical promise into real-world improvements at the bedside.

## 5 Advise: A National Advisory Council

### 5.1 The Need for Unified Standards

The Advise pillar advocates for a unified national voice to develop standards, share resources, and ensure equitable AI adoption across all emergency medicine practice settings. The ED is a uniquely demanding environment distinct from other clinical settings where AI tools developed and implemented to support clinical workflows and surface recommendations in the setting of acutely life-threatening conditions must be guided by clear and expert-informed best practices recommendations. Development of this guidance is best accomplished through collaborative, advisory leadership, and cross-organizational coordination.^33^^,^^55^ These recommendations ensure that AI tools are thoughtfully designed, rigorously evaluated, and responsibly implemented in ways that meet the unpredictable nature of emergency care.^30^^,^^31^ Hospitals can then use them as a conceptual compass for site-specific protocols without implying external oversight or regulatory authority. ACEP leadership and partner societies view this advisory council as a consensus imperative and the only scalable way to keep pace with AI deployment while maintaining specialty-wide standards.

### 5.2 A Diverse, Multiorganizational Voice

A national Emergency Medicine AI Advisory Council is well suited to developing best-practice recommendations, because it can launch under a concise charter, convene virtually, and iterate consensus statements quickly, while leveraging existing society resources and volunteer experts.^31^ Because the council operates under a concise charter with volunteer experts, it can issue nonbinding implementation toolkits quickly whereas hospitals share pilot results across academic, community, and critical-access settings.^31^^,^^33^

This advisory body should include representatives from ACEP, AAEM, SAEM, EMRA, and other stakeholders. It would issue nonbinding best-practice recommendations, shared language, and practical toolkits needed to help emergency physicians navigate the complexities of AI integration in their practice.^31^^,^^33^ By aligning on shared definitions, evaluation criteria, and implementation strategies, these organizations provide physicians, health systems, and industry partners with clear, consistent guidance and prevent the confusion that competing guidelines create.^35^

The group’s mandate would be to define a national strategy for AI in emergency medicine, with a focus on the following core areas:

1. Establishing core AI competencies for certification and accreditation for all emergency physicians
2. Developing and publishing an open, specialty-specific framework for evaluating AI tools
3. Maintaining a dynamic repository of training materials and implementation playbooks
4. Overseeing a network of pilot sites spanning academic centers, large community hospitals, and smaller critical access hospitals. Engaging these various institutions relevant to emergency care provides the foundation for the Council to develop guidelines that reflect the full spectrum of emergency medicine practice.

### 5.3 Advocating for Our Specialty

Without strong internal leadership to articulate consensus guidance, regulators, payors, or large technology firms will impose requirements that overlook bedside realities, so emergency physicians must stay at the helm to ensure AI improves patient outcomes.^24^^,^^40^^,^^41^^,^^47^

## 6 Conclusion

The integration of AI into emergency medicine is already underway across triage, imaging, clinical decision support, and operations.^4^, ^5^, ^6^, ^7^, ^8^, ^9^, ^10^, ^11^, ^12^, ^13^, ^14^, ^15^, ^16^, ^17^, ^18^, ^19^^,^^25^^,^^56^^,^^57^ To keep pace, clinicians need coordinated tools, training, and governance. This expert consensus opinion delivers 3 actions: standardize education from residency through ongoing CME so every physician shares a core competency baseline;^36^^,^^37^ deploy a clinician-led vetting framework that filters safe, effective tools from unvalidated products;^24^^,^^46^^,^^51^ and establish a national advisory body that issues shared terminology, best-practice guidance, and resource repositories without adding regulatory drag.^31^, ^32^, ^33^, ^34^, ^35^ Taken together, these three pillars—Educate, Evaluate, and Advise—ensure AI improves decision-making, supports equity, and protects patient outcomes.

## Funding and Support

By *JACEP Open* policy, all authors are required to disclose any and all commercial, financial, and other relationships in any way related to the subject of this article as per ICMJE conflict of interest guidelines (see www.icmje.org). The authors have stated that no such relationships exist.

## Declaration of Generative AI and AI-Assisted Technologies in the Writing Process

The authors used generally available large language models to assist with editorial refinement. All content was reviewed, verified, and approved by the authors, who take full responsibility for the accuracy, integrity, and originality of the work.

## Conflict of Interest

All authors have affirmed they have no conflicts of interest to declare.
